# Bioinformatic Characterization of Genes That Are Correlated to the Progression of Breast Cancer to Breast Cancer Brain Metastasis

**DOI:** 10.1002/cnr2.70360

**Published:** 2025-10-07

**Authors:** Mageshree Pillay, Oliver Tendayi Zishiri

**Affiliations:** ^1^ Discipline of Genetics, School of Life Sciences College of Agriculture, Engineering and Science, University of KwaZulu‐Natal Durban South Africa

**Keywords:** breast neoplasms, breast tumors, survival

## Abstract

**Background:**

The incidence of breast cancer is escalating into millions of cases annually all over the world with hundreds of thousands of deaths recorded each year. It has been well established that breast cancer is caused by both genetic and non‐genetic factors. However, there is a paucity of information on breast cancer that metastasizes to the brain. The molecular process of carcinogenesis in breast cancer brain metastasis (BCBM) is yet to be fully characterized.

**Aims:**

It is crucial to identify genes linked with breast cancer brain metastasis development and prognosis. This study sought out to decipher putative pathogenic and predictive genes in BCBM using bioinformatic analysis of public datasets.

**Methods and Results:**

The bioinformatic analysis utilized the GSE125989, GSE191230 and GSE52604 datasets. GEO2R was used for the identification of DEGs. Venn was employed to identify the common up‐regulated and down‐regulated genes. The STRING website was used to create the protein‐protein interaction (PPI) network of the DEGs, which was then represented using Cytoscape. A Kaplan–Meier (KM) plotter was used to conduct the hub gene survival analysis. Validation of the hub genes was carried out using UALCAN. The heat map was then visualized using Fun Rich. The tumor infiltrating analysis was carried out using TIMER. Using DAVID, the GO and KEGG analyses were conducted. The structure of the hub genes was obtained from the human protein atlas. A total of 4 DEGs was identified. A PPI network was developed, one significant module was identified, and 3 clusters were selected. Ten hub genes were discovered using Cytoscape‘s MCC ranking technique. Ten hub genes (*IL6, INS, TNF, PPARG, PPARA, SLC2A4, PPARGC1A, IRS1, LEP* and *ADIPOQ*) were all associated with the progression of BCBM.

**Conclusion:**

The study‘s findings revealed that the hub genes investigated could be possibly vital genes in determining the molecular mechanism of BCBM.

## 1 Introduction

Breast cancer is becoming increasingly prevalent in almost every part of the world, with industrialized countries having the greatest prevalence. There are an estimated 2.3 million new cancer cases and 685 000 cancer deaths each year [1]. The majority of all occurrences worldwide occur in affluent nations. This tendency is primarily attributable to the so‐called Western lifestyle, which is connected with unhealthy eating habits, excessive alcohol consumption, dependence on nicotine, elevated stress levels, and limited physical activity [2]. Breast cancer frequently originates in the cells that coat the ducts, the tubes that transport milk via the glands to the nipple [3].

The consistent advancement in both screening mammography and care is ascribed to the considerable drop in mortality caused by breast cancer in first‐world nations between 1975 and 2000 [4]. The likelihood of an individual getting breast cancer is often correlated to a range of genes in various pathways, such as the p53 signaling pathway, as well as with lifestyle, demographic, and nutritional variables. Histological grade, condition of the lymph nodes, and the magnitude of the tumor, common immunohistochemistry tests, and the state of hormone receptors composed of estrogen receptors (ER) and progesterone receptors (PR) are all used in the traditional categorization of breast cancer [5]. Genetic susceptibility and cellular adaptation mechanisms drive tumor cell growth in the brain microenvironment, which is greatly reliant on interactions between brain‐resident cells and tumor cells. While brain metastasis (BM) is much less prevalent in luminal A and B breast cancer, it is largely seen in TNBC (triple negative breast cancer) and HER2+ (Human Epidermal growth factor Receptor 2‐positive) breast cancer [6].

A paucity of information on breast cancer that metastasizes to the brain exists. Therefore, this study has the potential to contribute to the current hiatus. Although a plethora of information and treatment strategies exist for cancer, targeted gene therapies may prove to be extremely beneficial to breast cancer patients, and they may hopefully aid in mitigating the progression of breast tissue from becoming cancerous and decrease the progression of breast cancer to distant organs such as the brain. Discovering hub genes will greatly aid this cause; therefore, an investigation such as this study will be a significant contribution to the existing body of knowledge on breast cancer and breast cancer brain metastasis.

The incidence of brain metastasis is increasing because of combinations of improved and earlier screening/detection as well as more effective systemic chemotherapies [7, 8]. Improved therapeutic strategies for controlling primary tumors have resulted in longer survival times but have also raised the risk of late brain metastases. Despite the advent of new targeted medications, BM continues to be a deadly cancer consequence. Those suffering from BM have a dismal prognosis due to the lack of adequate long‐term therapy, thereby reducing an individual's rate of survival [9, 10]. However, progress in research on effective treatments is still modest, owing to the limited standard therapy options for brain metastases [11, 12]. Therefore, it is essential to find sensitive markers as new targets for improving the likelihood of survival for individuals with breast cancer brain metastasis [11]. To precisely anticipate recurrence variables for breast cancer brain metastasis and reduce the risk of its recurrence, this study implemented bioinformatic analysis to evaluate and discover therapeutic genes of interest for breast cancer brain metastasis by utilizing 3 GEO datasets.

## 2 Methodology

### 2.1 Datasets

In order to identify datasets that included both breast cancer and breast cancer brain metastasis tissue samples, the Gene Expression Omnibus (GEO) repository (https://www.ncbi.nlm.nih.gov/geo/) was employed. Subsequently, the datasets were chosen according to their sample count. After identifying datasets with more than 10 samples, they were then reviewed to determine whether they could be used for this investigation. The three datasets, GSE125989, GSE191230, and GSE52604, were then selected from the NCBI‐GEO data repository (https://www.ncbi.nlm.nih.gov/geo/).

The GSE125989 dataset was entitled “Prinary breast cancers and brain metastasis” and was comprised of 16 breast cancer samples and 16 breast cancer brain metastasis samples from Japan [13]. The GSE191230 dataset was entitled “RNA‐seq analysis on human primary breast cancer tumor and distant metastasis tumor” and was comprised of 13 primary breast tumors and 7 distant metastases of which 5 were breast cancer brain metastasis and 2 were breast cancer lung metastasis [14]. Only the breast cancer brain metastasis samples were used during the analysis. This dataset originated in Singapore. The GSE52604 dataset was entitled “Breast Brain Metastasis, Non‐Neoplastic Brain, and Non‐Neoplastic Breast Gene Expression” and originated from the USA, and was comprised of 35 Breast Brain Metastasis samples, 10 Non‐Neoplastic Brain samples, and 10 Non‐Neoplastic Breast samples [15]. The dataset platform for the GSE125989, GSE191230, and GSE52604 was the GPL571 [HG‐U133A_2] Affymetrix Human Genome U133A 2.0 Array, GPL20795 HiSeq X Ten ( *Homo sapiens* ), and the GPL6480 Agilent‐014850 Whole Human Genome Microarray 4x44K G4112F respectively.

### 2.2 Identification of Differentially Expressed Genes (DEGs)

Differential gene expression analysis is crucial for identifying genes with significant activity differences between primary tumors and brain metastases. These genes may play key roles in metastatic progression and serve as potential biomarkers or therapeutic targets.

GEO2R (https://www.ncbi.nlm.nih.gov/geo/geo2r/) with the criteria of adjusted *p*‐value < 0.05 was used for the detection of differentially expressed genes that exist between the cancerous breast tissue samples and the breast cancer brain metastasis tissue samples in each dataset, respectively [16]. Benjamini‐Hochberg correction was utilized to control false discovery rates, and log transformation was applied.

Volcano plots and u‐maps for each dataset were obtained after the GEO2R analysis was completed. Thereafter, the top 250 DEGs were downloaded and edited using Excel and filtered using Bash scripting on the Ubuntu platform for up‐regulated genes and down‐regulated genes using the following inclusion criteria: Up‐regulated genes—log2 FC (fold change) ≥ 2 and down‐regulated genes—log2 FC (fold change) ≤ −2.

An online tool (http://bioinformatics.psb.ugent.be/webtools/Venn/) was used to distinguish the common differentially expressed genes between the up‐regulated and down‐regulated genes, respectively, using Venn diagrams [17].

### 2.3 The Network Analysis and Visualization of the Protein–Protein Interaction (PPI)

Single genes seldom regulate biological processes; therefore, building a PPI network enables us to determine the manner in which DEGs interact within cellular pathways. This aids in identifying significant gene modules that promote metastasis.

An online analysis tool called String (https://string‐db.org/) was employed for the generation of the protein–protein interaction network diagram for the DEGs that were identified [18]. A confidence of 0.4 was used. The network was expanded until the expected number of edges exceeded 1000.

The network was thereafter imported onto Cytoscape (https://cytoscape.org/), which was utilized for the visualization of the molecular networks [19]. The plug‐in network analyzer tool was installed via Cytoscape and used to analyze the network. Thereafter, the plug‐in MCODE (Molecular Complex Detection) was installed and used to analyze the network using the following criteria: Haircut on, Node score cut‐off = 0.2, K‐Core = 2 [20]. The top 3 clusters with the highest score were then selected. The cytoHubba plug‐in app was then installed, and the nodes were ranked by MCC to find the top 10 genes.

### 2.4 Hub Gene Survival Analysis

Survival analysis links molecular findings to clinical outcomes. Therefore, by analyzing the prognostic value of hub genes, we assess whether gene expression levels correlate with patient survival, thereby validating their potential as biomarkers.

A Kaplan–Meier plot (https://kmplot.com/analysis/index.php?p=service&cancer=breast) was carried out [21]. This plot was used to determine the recurrence‐free survival (RFS) for breast cancer patients for each gene. This analysis was carried out against 2032 patients' data. The plot was also used to determine the distant metastasis‐free survival, which was carried out against 958 patients. The log‐rank *p*‐values were calculated with a 95% confidence interval, and the hazard ratio was also calculated. This analysis also shows the probability survival differences for high and low expression groups.

### 2.5 Hub Genes Validation Based on Different Pathological Features

Gene expression may not accurately represent biological impact on its own. Therefore, hub genes are validated across cancer subtypes using clinical datasets (TCGA) by evaluating promoter methylation patterns.

The methylation analysis makes it possible to compare different disease stages, giving insights into how primary breast cancer progresses to brain metastases. These insights can be used to guide therapeutic approaches and clinical management.

UALCAN (http://ualcan.path.uab.edu/analysis.html) was utilized to perform the promoter methylation levels analysis and validation of the 10 hub genes using the cancer genome atlas (TCGA) [22, 23]. The analysis was conducted based on the BRCA samples. A further analysis was carried out based on the different major subclasses.

### 2.6 Heat Map Visualization

Understanding tissue‐specific expression of hub genes can provide insight into their physiological functions and relevance to specific organs or cell types, potentially explaining organotropic metastasis.

Fun Rich (http://www.funrich.org/) was used to obtain the heatmap of the 10 hub genes using the human proteome database [24, 25, 26].

### 2.7 Tumor Infiltration Analysis

Immune cell infiltration is critical to cancer progression and metastasis. By correlating hub gene expression with immune infiltration, it is assessed whether these genes are involved in shaping the tumor microenvironment.

The web‐based application TIMER (https://cistrome.shinyapps.io/timer/), which features samples from various cancer types accessible via the TCGA database was utilized to explore the relationship between the expression of specific hub genes and tumor‐infiltrating immune cells (B cells, CD4+ T cells, CD8+ T cells, neutrophils, macrophages, and dendritic cells) [27, 28, 29].

### 2.8 Gene Ontology (GO) and the Analysis of the KEGG Pathway

GO and KEGG analyses enable functional annotation of genes and the identification of key biological processes and pathways enriched among the hub genes, providing mechanistic insight into metastasis.

For the identification of the gene ontology pathway for the top 10 hub genes.

- Biological processes.
- Molecular function.
- Cellular component.

DAVID (https://david.ncifcrf.gov/) was employed [30, 31, 32].

For the KEGG pathway analysis, DAVID (https://david.ncifcrf.gov/) was also used.

### 2.9 The Hub Gene Structure

Visualization of the 3D structure of hub gene products provides information about their molecular function and potential druggability, which aids in efforts to target them therapeutically later on.

The protein structure for each hub gene was obtained from the Human Protein Atlas (https://www.proteinatlas.org/) [33].

## 3 Results

### 3.1 Identification of Differentially Expressed Genes (DEGs)

**FIGURE 1:**
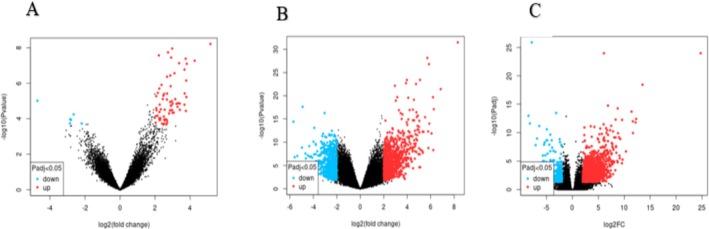
Volcano plots for the (A) GSE125989, (B) GSE52604, and (C) GSE191230 datasets respectively.

The volcano plots in Figure 1 display the statistical significance versus magnitude of change for each dataset, GSE125989, GSE52604, and GSE191230, respectively. The red on the volcano plot represents the up‐regulated genes, and blue represents the down‐regulated genes. The 750 differentially expressed genes (the top 250 in each dataset) were do**w**nloaded from GEO2R for the 3 datasets (GSE125989, GSE52604, and GSE191230) respectively.

The GSE125989 dataset comprised of 6 down‐regulated genes and 52 up‐regulated genes. The GSE52604 dataset comprised of 41 down‐regulated genes and 83 up‐regulated genes. The GSE191230 dataset comprised of 33 down‐regulated genes and 207 up‐regulated genes. The up‐regulated genes were within the threshold |log2 (FC)| ≥ 2, and the down‐regulated genes were within the threshold |log2 (FC)| ≤ −2, and both within the criteria of adjusted *p*‐value < 0.05.

**FIGURE 2:**
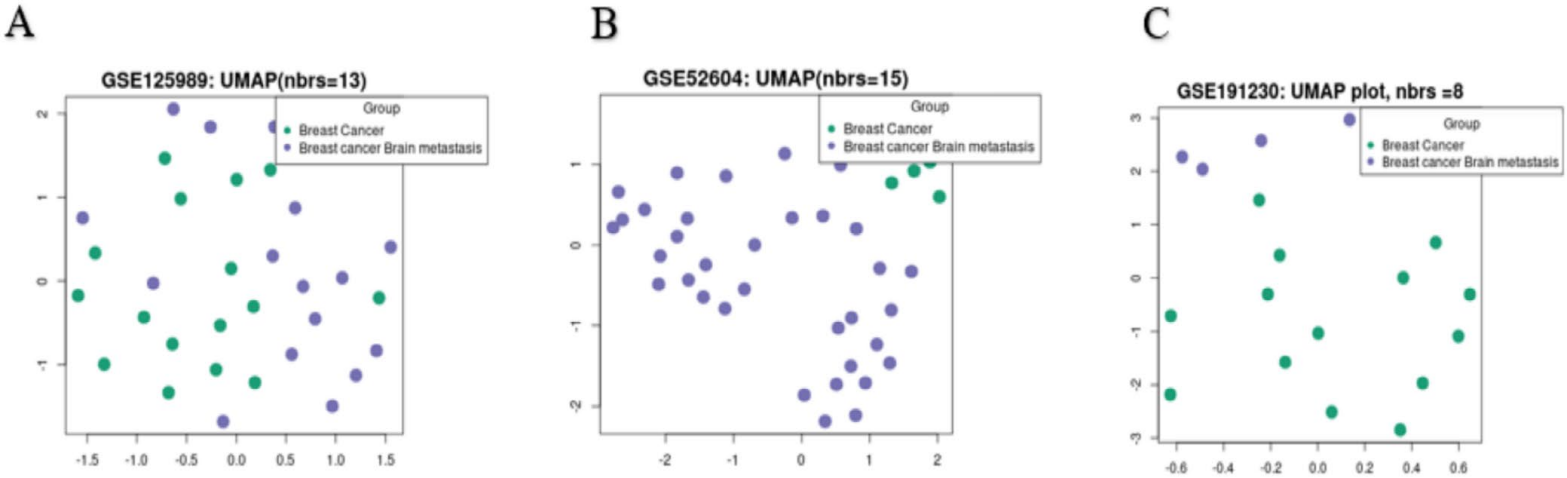
UMAP for the (A) GSE125989, (B) GSE52604, and (C) GSE191230 datasets respectively.

**FIGURE 3:**
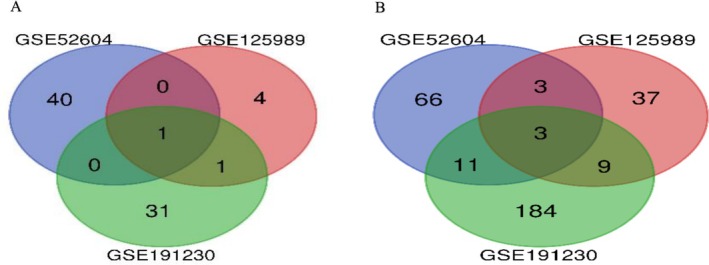
Venn diagram (A) overlapping down‐regulated genes (B) overlapping up‐regulated genes respectively.

In Figure 2A–C, the Uniform Manifold Approximation and Projection (UMAP) plot shows the relation between the samples in the groups. In Figure 2A–C, the number of nearest neighbors is 13, 15, and 8, respectively. In Figure 3A,B the Venn diagrams show us the common (overlapping) differentially expressed genes between the datasets; there is 1 common DEG between the down‐regulated genes and 3 common DEGs between the up‐regulated genes. In Table 1, the common (overlapping) differentially expressed genes among the datasets can be seen.

**TABLE 1:** The overlapping (common) genes between the datasets.

Gene regulation	Dataset names	Gene symbol
Down regulated	GSE125989	ZIC1
	GSE191230	
	GSE52604	
Down regulated	GSE125989	GFAP
	GSE191230	
Up regulated	GSE125989	COL15A1; JCHAIN; ADIPOQ
	GSE191230	
	GSE52604	
Up regulated	GSE125989	DPT; CILP; ADH1B; FABP4; PDGFRA; IGKC; CCL19; CTSK; CD3E
	GSE191230	
Up regulated	GSE191230	IRF4; CTSG; TBX5; POU2AF1; GNG2; TBX5‐AS1; TNFRSF8; COL6A6; SEMA3D; FZD10‐AS1; SRPX
	GSE52604	
Up regulated	GSE125989	MMP2; F2RL2; COL14A1
	GSE52604	

### 3.2 Protein–Protein Interaction (PPI) Network Analysis and Visualization

**FIGURE 4:**
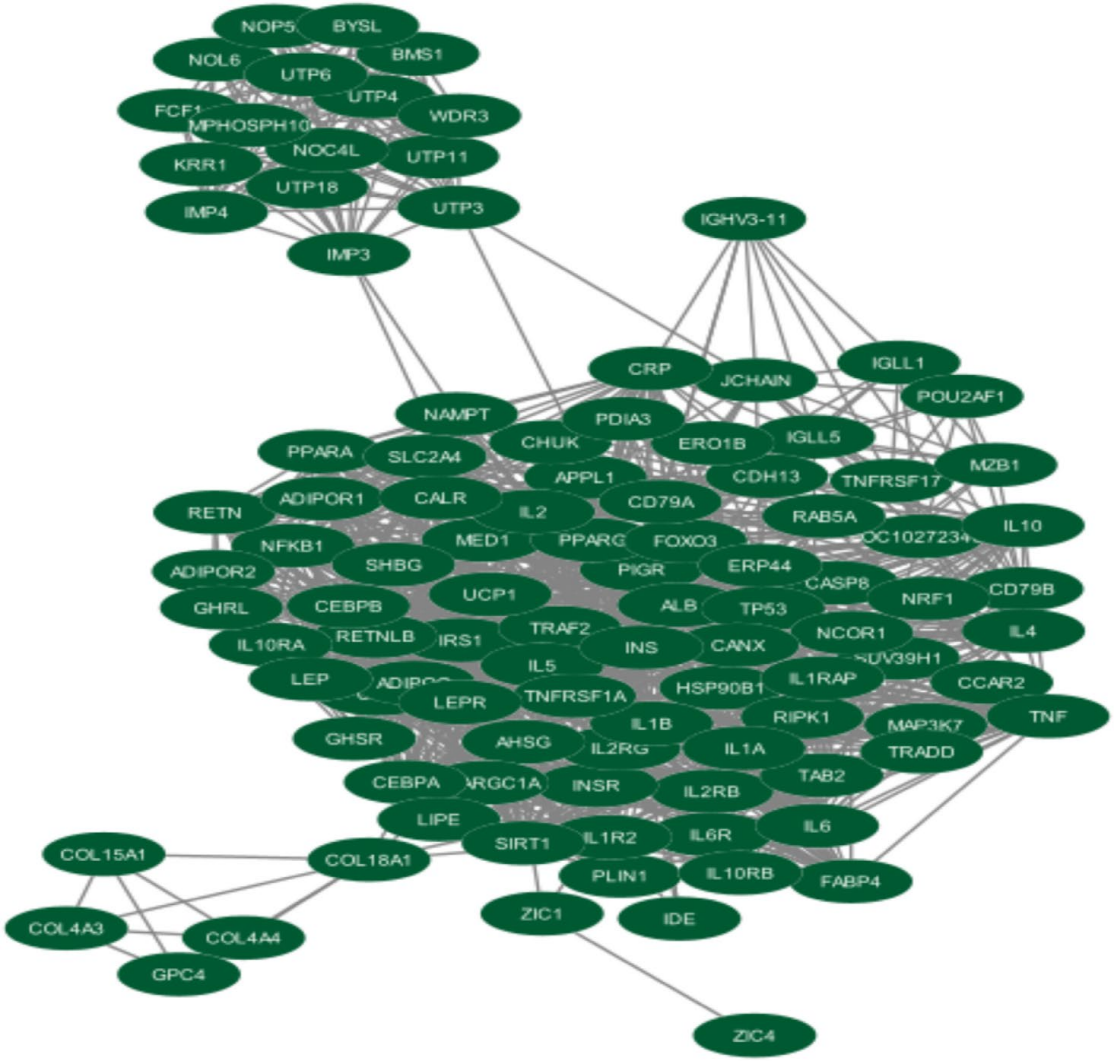
The protein–protein interaction network of the 4 differentially expressed genes.

Four [4] differentially expressed genes were identified between the 3 datasets (GSE125989, GSE191230, and GSE52604). The STRING database was used for the generation of the protein–protein interaction network, and it was visualized by Cytoscape. In Figure 4, the protein–protein interaction of the 4 differentially expressed genes (up and down‐regulated) can be seen with a medium confidence ≥ 0.4, and the interaction revealed that there were 104 nodes with 1056 edges and an average node degree of 20.3. The protein–protein interaction enrichment *p*‐value was < 1.0e‐6.

**FIGURE 5:**
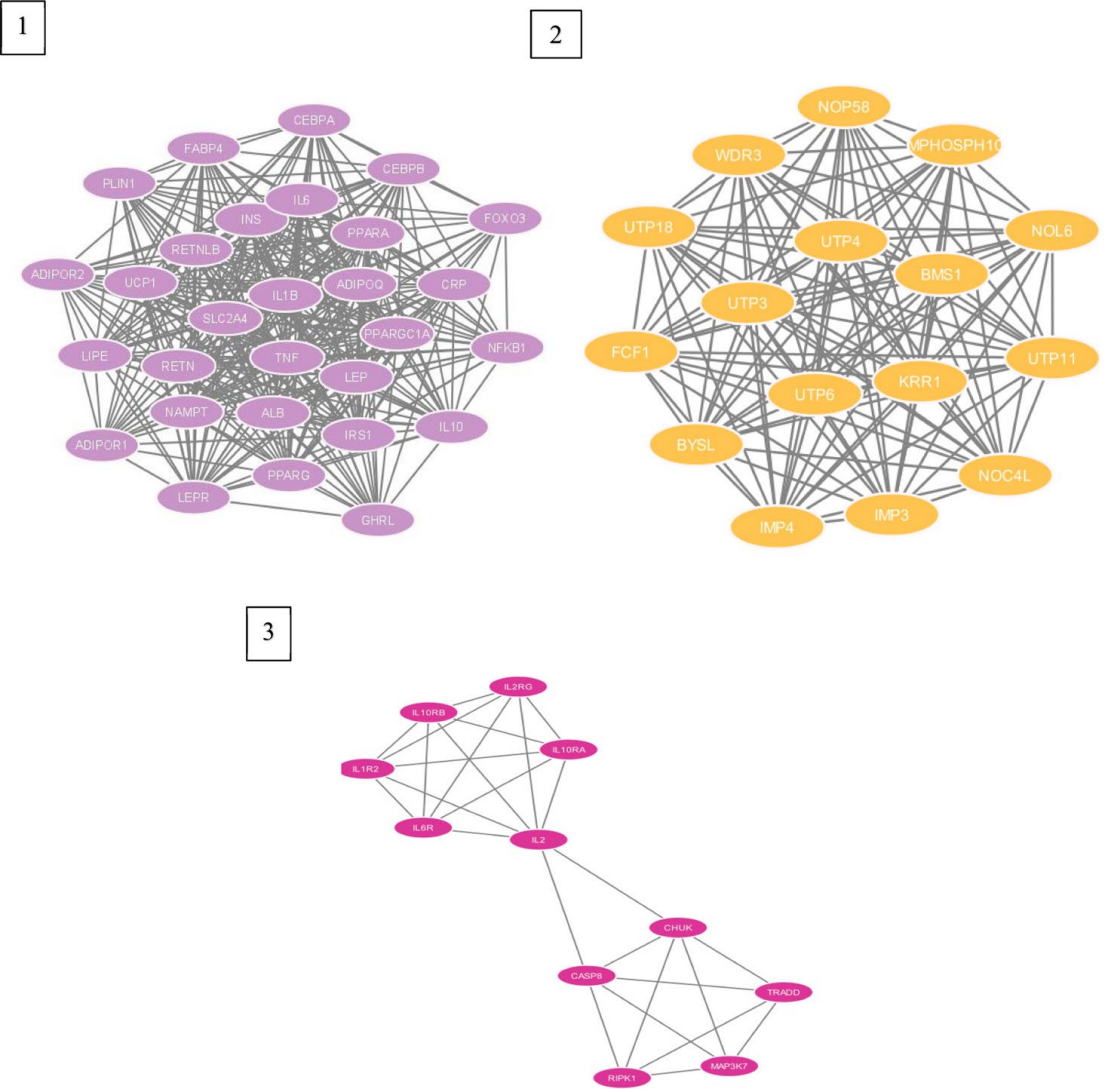
Significant clusters from the protein–protein interaction network that was obtained using the MCODE app, (1) MCODE score: 23.818, (2) MCODE score: 19.447, (3) MCODE score: 11.059.

**FIGURE 6:**
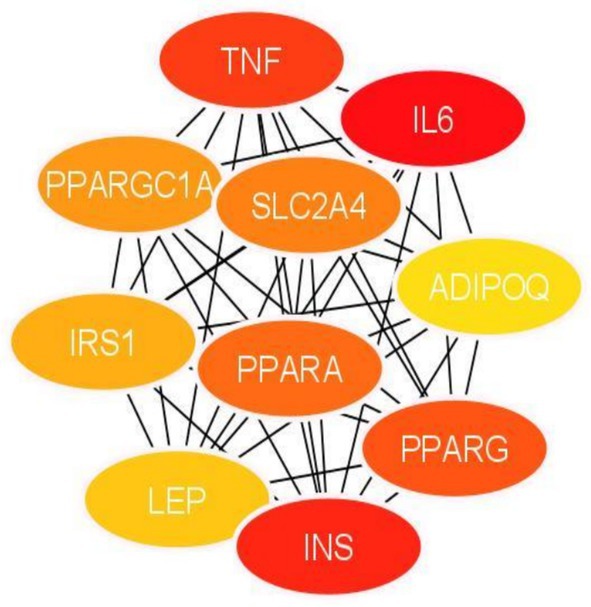
The top 10 hub genes identified via cytoHubba from the network of protein–protein interactions, ranked by MCC.

Figure 5 shows the significant clusters that were identified using the MCODE app with a score that exceeded 10. Figure 5A Cluster 1 has 29 nodes, 348 edges and a score of 24.857. Figure 5B Cluster 2 has 16 nodes, 120 edges, and a score of 16.00. Figure 5C Cluster 3 has 11 nodes, 27 edges, and a score of 5.400. The top 10 hub genes (Figure 6) were identified using a plug‐in app called cytoHubba in Cytoscape and ranked by using the MCC (maximal clique centrality) method as it attains the most crucial proteins from the high‐degree proteins as well as the low‐degree proteins in the top‐ranked list [34].

The top 10 hub genes (Figure 6) were found in Cluster 1, with the ranking score displayed from highest to lowest, with the red color indicating the higher degree score and yellow the lowest degree score. In Table 2, the top 10 hub genes with their respective ranking scores can be seen.

**TABLE 2:** The top 10 hub genes in the network ranked by the MCC method.

Rank	Hub gene
1	IL6
2	INS
3	TNF
4	PPARG
5	PPARA
6	SLC2A4
7	PPARGC1A
7	IRS1
9	LEP
10	ADIPOQ

### 3.3 The Survival Analysis of the Top 10 Hub Genes

The Kaplan–Meier plotter was used to determine the prognostic value of the 10 hub genes. Each of the top 10 hub genes showed potential for survival prediction based on their expression. Patients with breast cancer had their recurrence‐free survival and distant metastatic‐free survival (low vs. high) calculated based on the expression level of each hub gene.

**FIGURE 7:**
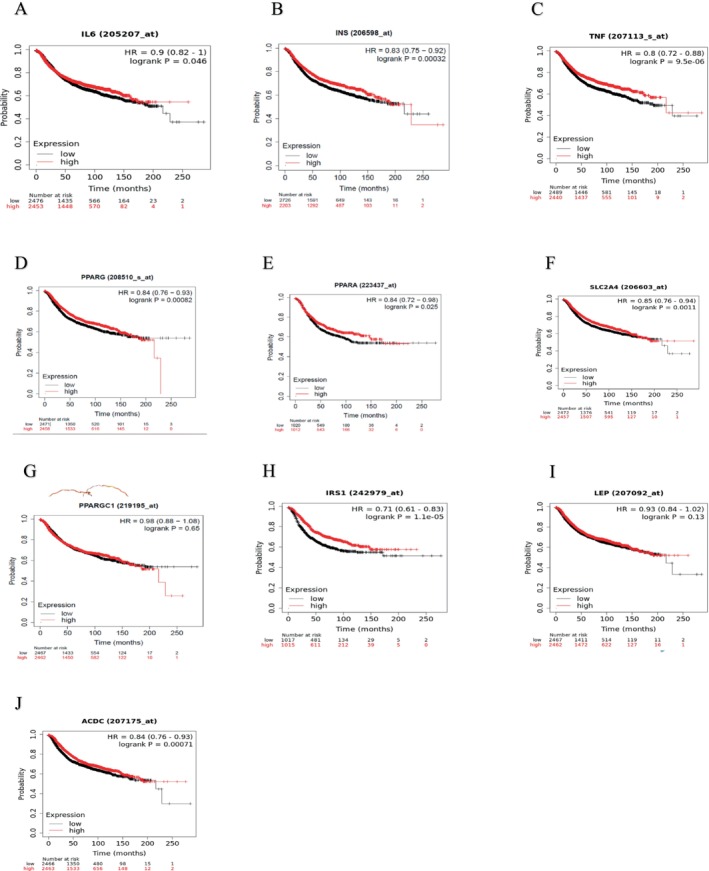
The Kaplan Meier curve for recurrence free survival (A) IL6, (B) INS, (C) TNF, (D) PPARG, (E) PPARA, (F) SLC2A4, (G) PPARGC1A, (H) IRS1, (I) LEP, (J) ADIPOQ.

The red represents a high expression, and the black a low expression on the Kaplan Meier curves. As can be seen in Figure 7A–J *IL6* HR = 0.9 (0.82–1) *p* = 0.046, *INS* HR = 0.83 (0.75–0.94) *p* = 0.00032, *TNF* HR = 0.8 (0.72–0.88) *p* = 9.5e‐06, *PPARG* HR = 0.84 (0.76–0.93) *p* = 0.00082, *PPARA* HR = 0.84 (0.72–0.98) *p* = 0.025, *SLC2A4* HR = 0.85 (0.76–0.94) *p* = 0.0011, *IRS1* HR = 0.71 (0.61–0.83) *p* = 1.1e‐05, *ADIPOQ* HR = 0.84 (0.76–0.93) *p* = 0.00071 was related to a poor recurrence‐free survival.

In Figure 7G,I, *PPARGC1A* HR = 0.98 (0.88–1.08) *p* = 0.65, *LEP* HR = 0.98 (0.84–1.02) *p* = 0.13 was not particularly associated with recurrence‐free survival.

**FIGURE 8:**
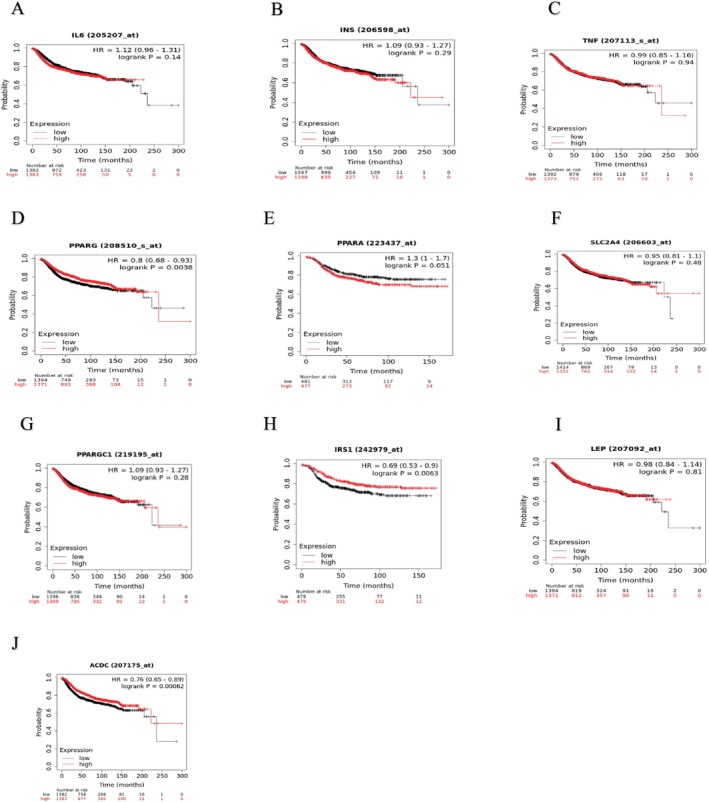
The Kaplan Meier curve for distant metastatic free survival (A) IL6, (B) INS, (C) TNF, (D) PPARG, (E) PPARA, (F) SLC2A4, (G) PPARGC1A, (H) IRS1, (I) LEP, (J)ADIPOQ.

As can be seen in Figure 8D,H,J *PPARG* HR = 0.8 (0.68–0.93) *p* = 0.0083, *IRS1* HR = 0.69 (0.53–0.9) *p* = 0.0063, *ADIPOQ* HR = 0.76 (0.65–0.89) *p* = 0.00062 was related to poor distant metastatic‐free survival.

In Figure 8A–C,E–G,I, *IL6* HR = 1.12 (0.96–1.31) *p* = 0.14, *INS* HR = 1.09 (0.93–1.27) *p* = 0.29, *TNF* HR = 0.99 (0.85–1.16) *p* = 0.94, *PPARA* HR = 1.31 (1–1.7) *p* = 0.051, *SLC2A4* HR = 0.95 (0.81–1.1) *p* = 0.48, *PPARGC1A* HR = 1.09 (0.93–1.4) *p* = 0.28, *LEP* HR = 0.98 (0.84–1.14) *p* = 0.81 was not particularly associated with distant metastatic‐free survival.

In Table 3, the high and low expression cohorts can be seen for the 10 hub genes with median or upper quartile survival in recurrence‐free survival. In Table 4, the high and low expression cohorts can be seen for the 10 hub genes with median or upper quartile survival in distant metastatic‐free survival.

**TABLE 3:** The expression cohorts for Kaplan‐Meier curve for recurrence‐free survival.

Survival type	Hub gene	Low expression cohorts (months)	High expression cohorts (months)
Upper quartile	IL6	47	53.04
Upper quartile	INS	216.66	228.85
Median	TNF	191.21	216.66
Median	PPARG	41.39	58
Median	PPARA	36	43
Upper quartile	SLC2A4	42	58.52
Median	PPARGC1A	48.48	50
Median	IRS1	29.6	47
Upper quartile	LEP	46	53.1
Upper quartile	ADIPOQ	41.39	57.27

**TABLE 4:** The expression cohorts for Kaplan Meier curve for distant metastatic free survival.

Survival type	Hub gene	Low expression cohorts (months)	High expression cohorts (months)
Median	IL6	84	77.23
Median	INS	236.22	222.81
Median	TNF	222.81	236.22
Upper quartile	PPARG	222.81	236.22
Median	PPARA	N/A	N/A
Median	SLC2A4	78	86.99
Upper quartile	PPARGC1A	222.81	236.22
Upper quartile	IRS1	NA	NA
Upper quartile	LEP	79.46	78.02
Upper quartile	ADIPOQ	236.22	222.81

### 3.4 The Validation of Hub Genes Based on Its Different Pathological Features

The promoter methylation levels of the top 10 hub genes were explored using the UALCAN database, and the major breast cancer subclasses' promoter methylation levels were also explored, as tumor development is highly associated with the degree of methylation in the promoter regions.

**FIGURE 9:**
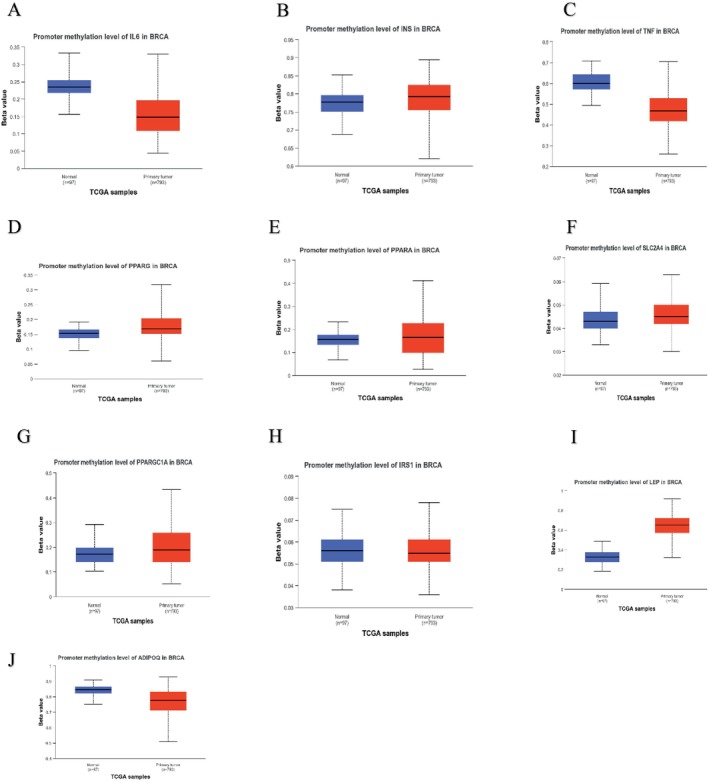
Promoter methylation levels based on different cancer types (A) IL6, (B) INS, (C) TNF, (D) PPARG, (E) PPARA, (F) SLC2A4, (G) PPARGC1A, (H) IRS1, (I) LEP, (J)A DIPOQ.

**FIGURE 10:**
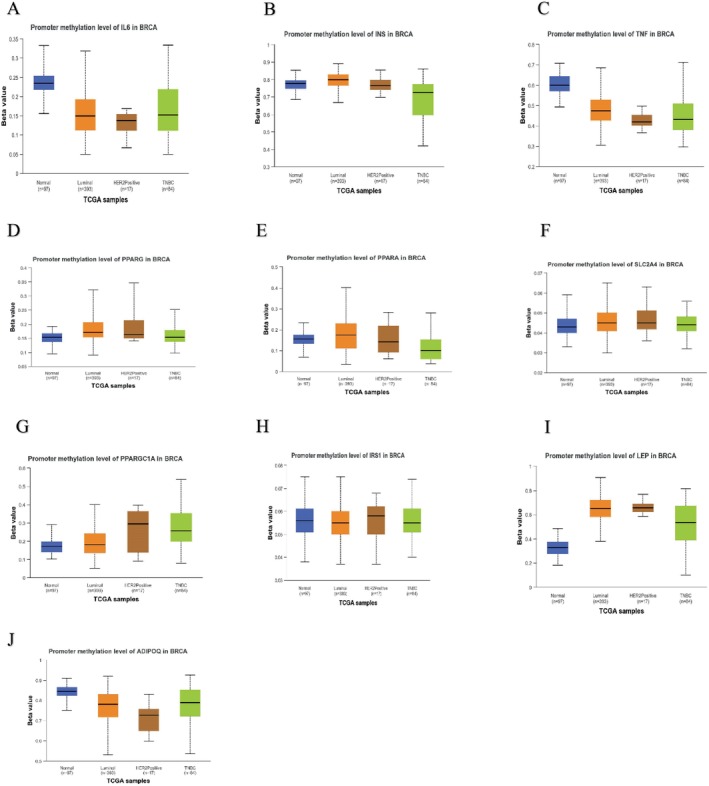
Promoter methylation levels for the different cancer subclasses (A) IL6, (B) INS, (C) TNF, (D) PPARG, (E) PPARA, (F) SLC2A4, (G) PPARGC1A, (H) IRS1, (I) LEP, (J) ADIPOQ.

The promoter methylation levels in *IL6* (Figure 9A), *TNF* (Figure 9C), and *ADIPOQ* (Figure 9J) primary tumors were considerably lower than normal. Based on the major breast cancer subclasses, *IL6* (Figure 10A) was found to have lower promoter methylation levels in the primary tumor compared to normal.

The promoter methylation levels in *INS* (Figure 9B), *PPARG* (Figure 9D), *PPARA* (Figure 9E), *SLC2A4* (Figure 9F), *IRS1* (Figure 9H), *PPARGC1A* (Figure 9G), and *LEP* (Figure 9I) were higher in primary tumors than in normal.

Based on the major breast cancer subclasses, *INS* (Figure 10B), *TNF* (Figure 10C), *PPARG* (Figure 10D), *PPARA* (Figure 10E), *SLC2A4* (Figure 10F), *IRS1* (Figure 10H), *PPARGC1A* (Figure 10G), *LEP* (Figure 10I), and *ADIPOQ* (Figure 10J) were found to have higher promoter methylation levels in the tumor breast cancer subclasses compared to normal.

Among the subclasses, *INS*, *PPARA*, *SLC2A4*, *IRS1*, and *LEP* were found to have higher promoter methylation levels in the Luminal breast cancer subclass. *TNF*, *PPARGC1A*, and *ADIPOQ* had a higher promoter methylation level in the TNBC subclass. *PPARG* had a higher promoter methylation level in the HER2‐Positive subclass.

### 3.5 Heat Map Visualization

**FIGURE 11:**
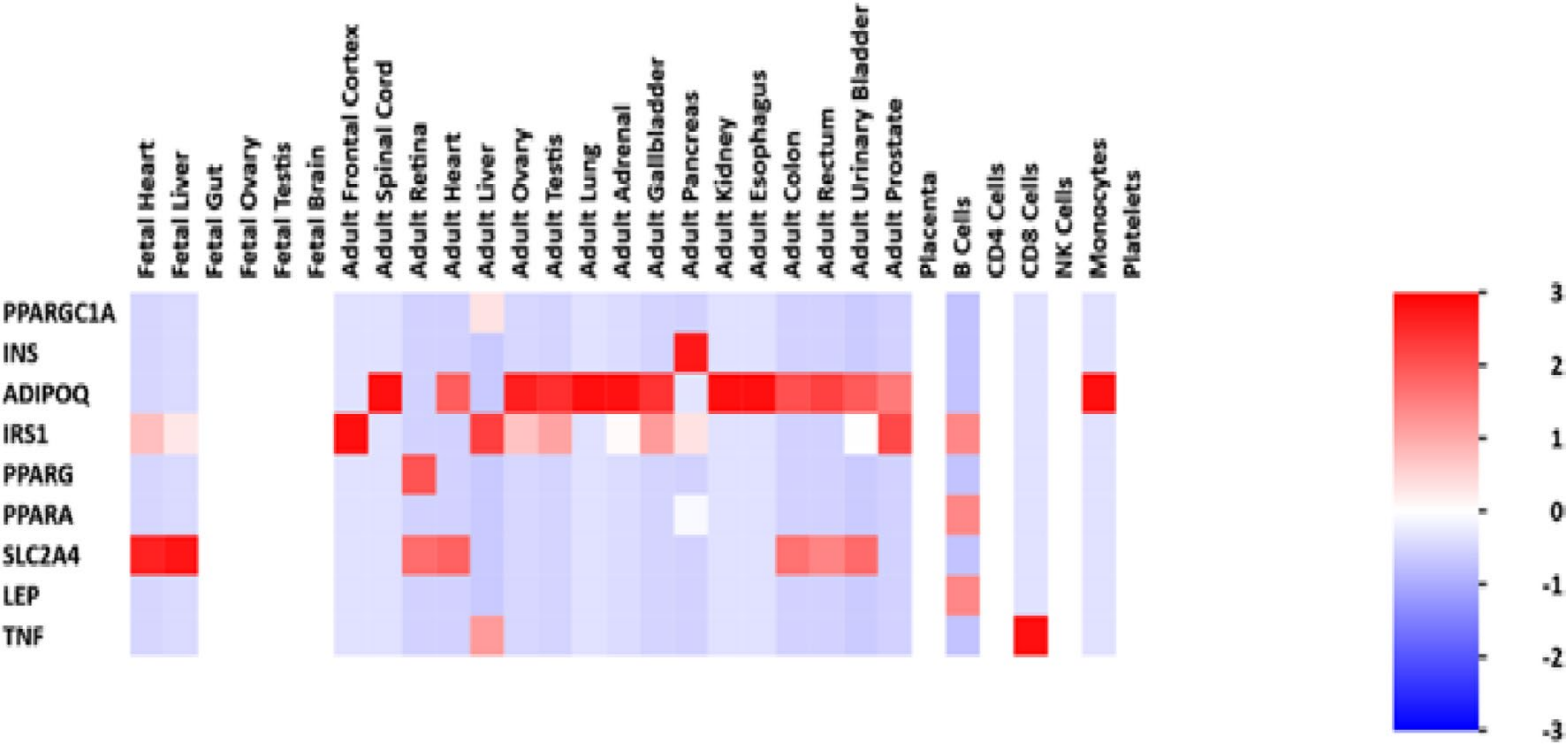
The heat map visualization of the top 10 hub genes.

The heatmap in Figure 11 shows other organs in which the hub genes identified in this study are prevalent. The *INS* hub gene is prevalent in the adult pancreas. *ADIPOQ* is highly prevalent in the adult spinal cord, ovary, testis, lung, adrenal, gallbladder, kidneys, esophagus, and monocytes and does show expression in the placenta, NK, and CD4 Cells. The *IRS1* is highly prevalent in the adult frontal cortex. The *SLC2A4* hub gene is seen to be highly prevalent (indicated by the darker shade of red) in the fetal heart and liver. The *TNF* hub gene is highly prevalent in CD8 Cells.

### 3.6 Tumor Infiltration Analysis

**FIGURE 12:**
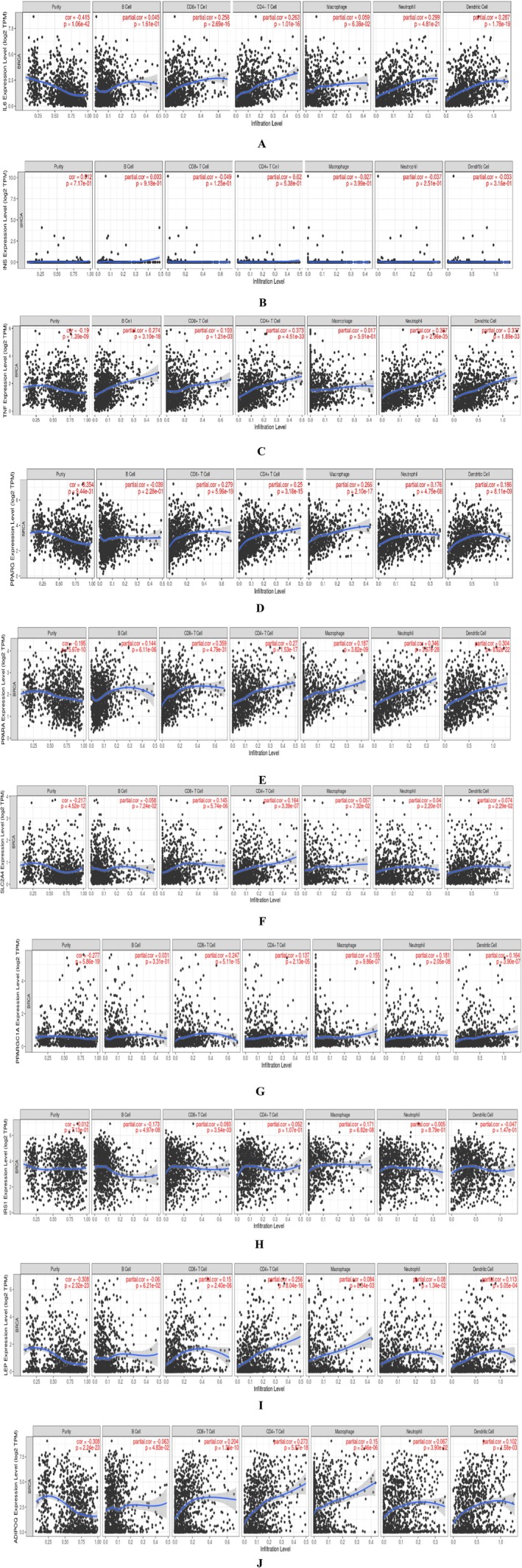
(A) The association between the IL6 expression and the 6 types of immunological infiltration cells. (B) The association between the INS expression and the 6 types of immunological infiltration cells. (C) The association between the TNF expression and the 6 types of immunological infiltration cells. (D) The association between the PPARG expression and the 6 types of immunological infiltration cells. (E) The association between the PPARA expression and the 6 types of immunological infiltration cells. (F) The association between the SLC2A4 expression and the 6 types of immunological infiltration cells. (G) The association between the PPARGC1A expression and the 6 types of immunological infiltration cells. (H) The association between the IRS1 expression and the 6 types of immunological infiltration cells. (I) The association between the LEP expression and the 6 types of immunological infiltration cells. (J) The association between the ADIPOQ expression and the 6 types of immunological infiltration cells.

In Figure 12A, *IL6* is positively linked with tumor purity. A strong link (*p* < 0.05) is found between the CD8+ T Cell, CD4+ T Cell, neutrophil, and dendritic cell. In Figure 12B, *INS* is negatively linked with tumor purity. In Figure 12C, *TNF* is positively linked with tumor purity. A strong connection is found between B Cell, CD8+ T Cell, CD4+ T Cell, neutrophil, and dendritic cells. In Figure 12D, *PPARG* is positively linked with tumor purity and has a strong association with CD8+ T Cell, CD4+ T Cell, macrophage, neutrophil, and dendritic cells. In Figure 12E, *PPARA* is positively linked with tumor purity. A strong association is found between the B Cell, CD8+ T Cell, CD4+ T Cell, macrophage, neutrophil, and dendritic cell. In Figure 12F, *SLC2A4* is positively linked with tumor purity and has a strong association with CD8+ T Cell, CD4+ T Cell, and dendritic cells. In Figure 12G, *PPARGC1A* is positively linked with tumor purity and has a strong association with CD8+ T Cell, CD4+ T Cell, macrophage, neutrophil, and dendritic cell. In Figure 12H, *IRS1* is negatively linked with tumor purity, but a strong association is found between the B Cell, CD8+ T Cell, and macrophage. In Figure 12I, *LEP* is positively linked with tumor purity and has a strong association with CD8+ T Cell, CD4+ T Cell, macrophage, neutrophil, and dendritic cell. In Figure 12J, *ADIPOQ* is positively linked with tumor purity and has a strong association with B Cell, CD8+ T Cell, CD4+ T Cell, macrophage, neutrophil, and dendritic cells.

### 3.7 Gene Ontology (GO) and the Analysis of the KEGG Pathway

The GO and KEGG pathway enrichment analysis was performed using David. The gene ontology analysis was done in 3 parts: (i) biological processes, (ii) cellular components, and (iii) molecular functions. The hub genes were predominately related to glucose homeostasis, negative regulation of lipid storage, cellular response to insulin stimulus, and response to ethanol (Table 5). The prominent cellular components that the hub genes were enriched in are the extracellular space, extracellular region, and cell surface (Table 6). The top identified molecular functions were hormone activity, cytokine activity, insulin‐like growth factor receptor binding, and insulin receptor binding (Table 7). From the KEGG analysis, it was revealed that the hub genes were enriched in the adipocytokine signaling pathway, insulin resistance, AMPK signaling pathway, and the longevity regulating pathway (Table 8).

**TABLE 5:** Gene ontology of the top 4 biological processes. GO items with a *p*‐value < 0.05.

ID	GO Terms	*p*	Genes
GO:0042593	Glucose homeostasis	4.7E‐12	ADIPOQ; IRS1; INS; IL6; LEP; PPARG; SLC2A4
GO:0010888	Negative regulation of lipid storage	5.7E‐9	IL6; LEP; PPARG; TNF
GO:0032869	Cellular response to insulin stimulus	1.0E‐7	ADIPOQ; IRS1; LEP; PPARG; SLC2A4
GO:0045471	Response to ethanol	1.3E‐7	ADIPOQ; LEP; PPARA; SLC2A4; TNF

**TABLE 6:** Gene ontology of the top 3 cellular components. GO items with a *p*‐value < 0.05.

ID	GO terms	*p*	Genes
GO:0005615	Extracellular space	7.3E‐3	ADIPOQ; INS; IL6; LEP; TNF
GO:0005576	Extracellular region	1.1E‐2	ADIPOQ; INS; IL6; LEP; TNF
GO:0009986	Cell surface	3.1E‐2	ADIPOQ; SLC2A4; TNF

**TABLE 7:** Gene ontology of the top 4 molecular function. GO items with a *p*‐value < 0.05.

ID	GO terms	*p*	Genes
GO:0005179	Hormone activity	1.2E‐3	ADIPOQ; INS; LEP
GO:0005125	Cytokine activity	3.5E‐3	ADIPOQ; IL6; TNF
GO:0005159	Insulin‐like growth factor receptor binding	7.6E‐3	IRS1; INS
GO:0005158	Insulin receptor binding	1.1E‐2	IRS1; INS

**TABLE 8:** KEGG pathways, enriched pathways with a *p*‐value < 0.05.

GO Terms	*p*	Genes
Adipocytokine signaling pathway	1.8E‐11	PPARGC1A; ADIPOQ; IRS1; LEP; PPARA; SLC2A4; TNF
Insulin resistance	2.8E‐10	PPARGC1A; IRS1; INS; IL6; PPARA; SLC2A4; TNF
AMPK signaling pathway	5.6E‐10	PPARGC1A; ADIPOQ; IRS1; INS; LEP; PPARG; SLC2A4
Longevity regulating pathway	1.3E‐6	PPARGC1A; ADIPOQ; IRS1; INS; PPARG

### 3.8 The Hub Gene Structure

**FIGURE 13:**
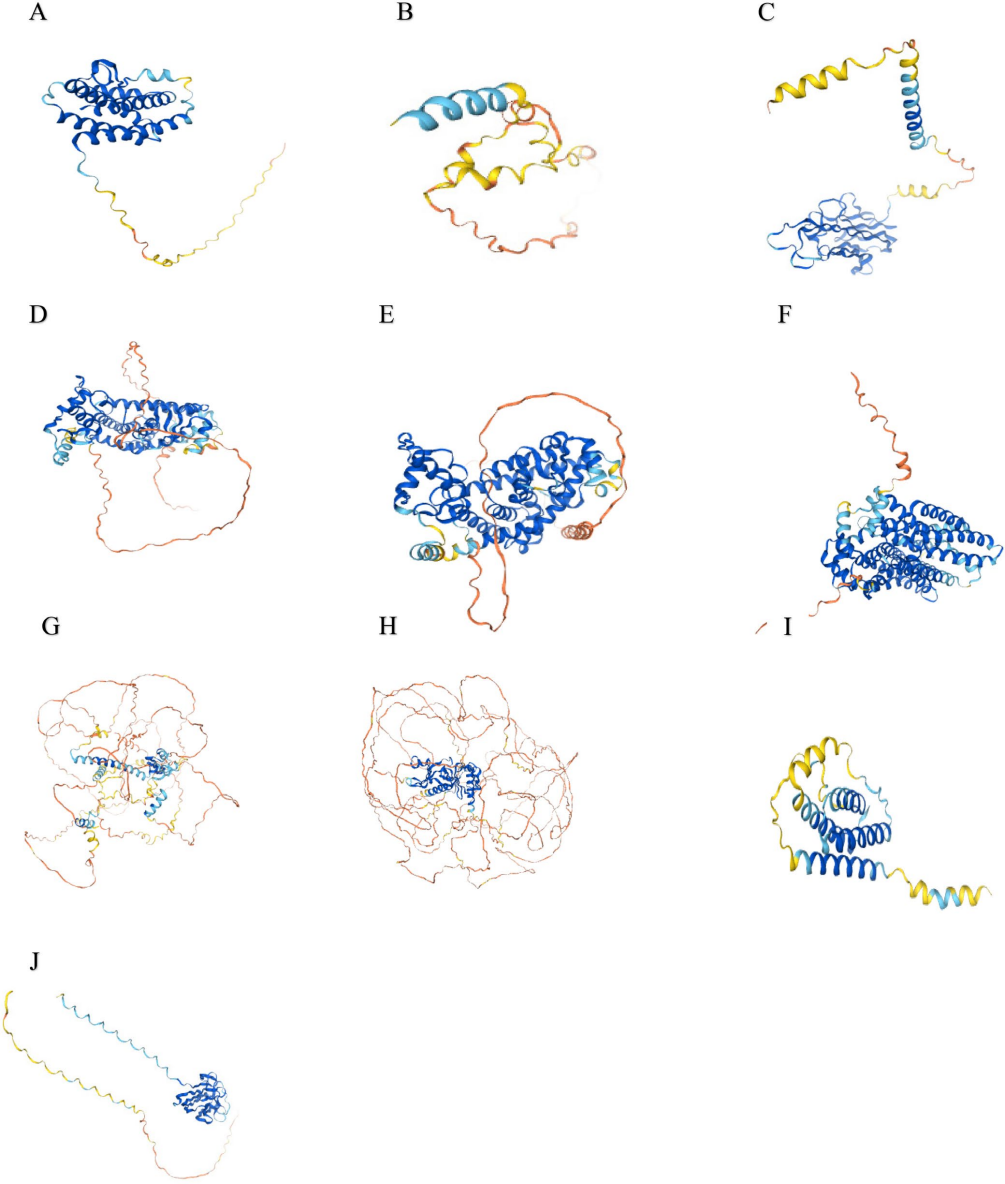
The protein structures of the top 10 hub genes (A) IL6, (B) INS, (C) TNF, (D) PPARG, (E) PPARA, (F) SLC2A4, (G) PPARGC1A, (H) IRS1, (I) LEP, (J) ADIPOQ.

The hub gene structure for the top 10 hub genes can be seen in Figure 13.

## 4 Discussion

Breast cancer has evolved into an enormous risk to world health due to the poor prognosis and quality of life it is linked with, as well as the significant potential for metastasis of malignant cells from the main tumor to distant organs [4, 35]. Breast cancer has a significantly high mortality rate, which is majorly due to there being inadequate screening methods that have a relatively high sensitivity as well as specificity [36]. Chemotherapy medications also have a rather difficult time passing through the blood–brain barrier; therefore, other therapies are needed to help increase the lifespan of patients with BCBM. Therefore, identifying hub genes can aid in early detection and the development of new treatment therapies. The development of next‐generation sequencing and microarray technology has also made it possible to analyze the genetic makeup of breast cancers and identify the course of the cancer.

In this study, three [3] GEO datasets were used for the identification and analysis of DEGs. The 750 DEGs were identified, and after further screening, four were found to be common among the three datasets. Thereafter, String was used for the construction of the PPI network. The PPI network was analyzed using Cytoscape, an in‐app that was employed for the identification of the most prevalent gene cluster (referred to as cluster 1). A further in‐app was employed called cytoHubba to identify the top 10 hub genes according to their MCC score. The top 10 hub genes were *IL6, INS, TNF, PPARG, PPARA, SLC2A4, PPARGC1A, IRS1, LEP*, and *ADIPOQ*.

As a member of the cytokine family of signaling molecules, interleukin‐6 (*IL‐6*) has been inferred in numerous studies as a potential regulator of aromatase activity and estrogen synthesis. Additionally, *IL‐6* may help prevent the development of aggressive metastases in secondary sites during initial adjuvant breast cancer therapy [37, 38]. It has further been discovered that the *IL‐6/IL‐6Ra* signaling mediates the cross‐talk that occurs between breast cancer metastasis and tumors; it was also found that cross‐talk significantly impacts treatment resistance and the relapse of cancer [39]. In this study, it was discovered that *IL‐6* was related to a poor recurrence‐free survival (Figure 7A), and it was discovered to be positively associated with tumor purity, thus indicating that it might aid in the spread and metastasis of tumors. This connection frequently reflects the larger inflammatory milieu that can facilitate the survival and growth of cancer cells. A potential treatment route to enhance outcomes for patients with certain malignancies is to target *IL‐6* or its signaling pathways.

It has recently been discovered that tumor‐secreted *IL‐6* increases the propensity for metastasis by training monocyte‐dendritic progenitors to prime distant organs for the growth of breast cancer [40]. Numerous studies have demonstrated that brain metastases provide a very bad prognosis for females with metastatic breast cancer. The key factor impacting the incidence and prognosis of BCBM is the subtype. Although the longevity of individuals with TN or HER2+ metastatic breast cancer is increasing, the development of brain metastases frequently reduces the patient's time to survival [41]. In this study, it was found that the promoter methylation levels were lower in the primary tumor when compared to normal breast tissue samples (Figure 9A). Thus, this may result in an increase in *IL‐6* expression since it is linked to inflammation and the development of tumors [42]. It was also discovered that the promoter methylation levels were higher for triple‐negative breast cancer compared to human epidermal growth factor receptor‐2 positive (Figure 10A). The distant metastatic‐free survival for *IL6* with a low expression was 84 months, and with high expression, it was 77.23 months (Table 4). Higher methylation levels in TNBC can change pathways involved in cell growth and survival and silence tumor suppressor genes, whereas HER2‐positive cancers are frequently characterized by unique molecular features brought on by overexpression of HER2 [43]. It is essential to comprehend these epigenetic modifications in order to create tailored treatments and enhance approaches to treating various forms of breast cancer.

Insulin (*INS*) is released by beta cells in the pancreatic islet and encourages a microenvironment that is dysfunctional toward the development and growth of breast cancer metastasis [44, 45]. Insulin is detected by insulin receptors that are found in numerous cells in the human body, including tumor cells. Numerous studies have demonstrated that people with insulin resistance and metabolic disorders such as type 2 diabetes have a greater chance of getting cancer as these disorders aid in the growth and development of dysregulating various oncogenic pathways that are regulated by cytokines [46, 47].

Hyperinsulinemia, which is an increased amount of insulin in the bloodstream, is said to be a predictive factor for breast cancer [48]. In Figure 7B, from the hub gene survival analysis, it was found that *INS* was linked to poor recurrence‐free survival in patients with a low expression cohort and a high expression cohort of 47 months and 53.04 months, respectively (Table 3). In Figure 9B, it was discovered that *INS* had higher promoter methylation levels in primary tumors compared to normal breast tissue samples. This could indicate the possibility that epigenetic changes contribute to the development of malignancies [49]. The *INS* gene may be silenced as a result of this high methylation, which may impact insulin signaling pathways that regulate tumor growth and metastasis [50].

*TNF* (Tumor necrosis factor) is a versatile, pro‐inflammatory cytokine that is released by inflammatory cells and is a part of various biological activities such as cell survival, proliferation, differentiation, and death [51, 52]. The *TNF* family includes *TNF alpha, TNF beta*, CD40 ligand, and various others [53]. *TNF* has been identified as a vital risk factor for carcinogenesis, progression, invasion, and metastasis; however, *TNF* signaling can play opposing roles in carcinogenesis and metastasis, and depending on the environment, the type of tumor, and the stage of tumor growth, it can either promote or hinder tumor progression, according to recent studies [54, 55]. Because of this dual function, treatment approaches that target the TNF system have had to be evaluated.

In Figure 10C, *TNF* was discovered to have a higher promoter methylation level in the TNBC subclass. In Figure 12C, *TNF* is positively linked with tumor purity with a strong connection between B Cell, CD8+ T cell, CD4+ T cell, neutrophil, and dendritic cell, thus indicating that changes to the epigenetic code may suppress its expression. TNF may play a role in regulating the immune system in the tumor microenvironment [56, 57]. It was also discovered that in the premetastatic brain, the upregulation of *S100A8/A9* and *SAA3* was linked to higher levels of *TNF alpha* [58].

Nuclear receptors in the peroxisome proliferator‐activated receptor (*PPAR*) are represented by the *PPARG* gene in humans [59]. The initiation of *PPARG* promotes the proliferation of cells and the progression of metastatic disease in the brain. Astrocytes are contributors of *PPAR* activators to the invading cancer cells due to their increased polyunsaturated fatty acid concentration. Brain metastatic lesions exhibit much greater levels of *PPARG* signaling in clinical samples [60, 61]. *PPARG* was related to poor distant metastatic‐free survival with a low‐expression cohort and a high‐expression cohort of 222.81 months and 236.22 months, respectively (Table 4). *PPARG* is expressed in many cells such as B cells, CD8+ T cells, CD4+ T cells, macrophages, neutrophils, and dendritic cells, as can be seen in Figure 12D, and studies have also found that it is expressed in adipocytes and smooth muscle cells [62]. PPARG activation in these immune cells has the potential to impact the inflammatory microenvironment within tumors, thus influencing tumor growth, metastasis, and therapeutic response [63, 64]. PPARG is being investigated as a possible therapeutic target for autoimmune illnesses and cancer immunotherapy due to its function in regulating immune responses [65, 66].

*PPARA* (peroxisome proliferator‐activated receptor alpha) was the first *PPAR* found, which is initiated by various peroxisome proliferators and is associated with numerous malignancies [67]. *PPARA* is typically engaged in the metabolism of glucose and lipids. It has recently been discovered that breast cancer is linked to metabolic pathways such as the de novo synthesis, fatty acid transport, and various others [68]. Through β‐oxidation, breast cancer tumors can take in and use free fatty acids from adjacent adipocytes as energy for tumor growth. *PPARA* is also associated with the proliferation and metastasis of breast cancer [69]. It was discovered that the *PPARA* genetic polymorphism rs4253760 was linked to postmenopausal breast cancer with a 2‐fold increase and was also seen in the adapted hierarchical models for the *PPAR*s SNPs [68, 70]. Monocytes express *PPARA*, which is increased as they develop into macrophages; this is supported by Figure 12E, where it is seen that *PPARA* has a strong association with macrophages. Macrophages in the tumor microenvironment play an important role in the promotion of malignancy and poor prognosis. Due to the rapid growth of tumor cells, macrophages face a shortage of certain nutrients, such as glucose [71, 72]. The regulation of inflammatory reactions is a function of *PPARA*. Elevated concentrations may impact macrophage polarization, thereby endorsing an anti‐inflammatory M2 phenotype that is linked to tumor advancement and tissue repair [73]. The expression of *PPARA* may improve macrophages' capacity to modulate immune responses, influencing their interactions with tumor cells and other immune cells.

As global DNA methylation analysis and various other DNA sequencing methods are used, it enhances the opportunity for the identification of various epigenetic changes that might be linked to tumor progression and oncogenesis [74, 75]. In Figure 10E, *PPARA* was discovered to have a higher promoter methylation level in the Luminal cancer subclass.

*SLC2A4* (Solute carrier family 2 member 4), the insulin‐responsive glucose carrier, is primarily located in adipocytes and muscle cells and is often referred to as *GLUT4* [76]. One of the primary biochemical processes that define tumor cells is glycolysis, which breaks down glucose into smaller molecules through glucose transport, which is mediated by GLUT [77]. The results of this study concur with the findings of other studies [78] that *SLC2A4* is a possible prognostic biomarker for the survival of breast cancer patients, as this study found that *SLC2A4* is related to a poor recurrence‐free survival for individuals with breast cancer when highly expressed (Figure 7F), with a low expression cohort and a high expression cohort of 42 months and 58.2 months, respectively (Table 3). However, it was not particularly associated with distant metastatic‐free survival. High *SLC2A4* expression could be a sign of improved glucose uptake by tumor cells, which would promote their rapid proliferation and survival [79]. Aggressive tumor behavior might arise from this metabolic adaptation. One potential therapeutic approach to prevent tumor growth and enhance patient outcomes is to target *SLC2A4*. Inhibiting the amount of glucose transported could limit the tumor's energy source [80].

The *PPARGC1A* (peroxisome proliferator‐activated receptor coactivator‐1 alpha) is a known regulator of cancer metabolism that is becoming an essential driver of metabolic pathways [81]. *PGC‐1A* stimulates nuclear receptors and transcription factors in breast cancer, including *PPAR* and numerous others, increasing mitochondrial biogenesis and OXPHOS and producing significant amounts of ATP needed for the development of tumors [82]. In metabolic pathways such as glycolysis, glutaminolysis, and various others, the biological functions of *PGC‐1A* were discovered even though *PGC‐1A* has primary biological significance for mitochondrial respiration [82, 83, 84].

*PGC‐1A* levels are quite susceptible to various environmental stimuli. In studies it has been shown that *PGC‐1A* can be regulated by oxygen and nutrition levels, which can vary significantly during tumor growth [85]. A strong correlation was discovered between the development of distant metastases and the expression of *PGC‐1A* in invasive cancer cells, as demonstrated by clinical investigations of human invasive breast tumors [86]. In this study, it was discovered that *PPARGC1A* was not particularly associated with distant metastatic‐free survival and had a low expression cohort and a high expression cohort of 222.81 months and 236.22 months respectively (Table 4).

*IRS1* (Insulin receptor substrate 1) is extensively expressed in breast cancer cell lines and has also been discovered in metastases by scRNAseq as it promotes the proliferation of tumors. *IRS1* is the primary docking protein in normal cells, where it binds and activates the insulin signaling system [87, 88]. During ligand activation, *IRS* (Insulin receptor substrates) are drawn to the IGF receptors (Type I), and they essentially control cell activity as they are responsible for transducing signals to downstream signaling chain reactions [89]. The main signaling molecule that is triggered by IGF‐I is *IRS1* in breast cancer cells. Increased tyrosine phosphorylation of *IRS1* by IGF‐I is linked to increased activation of mitogenic downstream signaling pathways [89, 90]. In a few studies, contradictory information was found regarding the role of IRS1 in patient prognosis in metastatic breast cancer.

From our observations and findings, it is suggested that a high expression of *IRS1* in invasive breast cancer with HR = 0.71 (0.61–0.83) and *p* = 1.1e‐05 was associated with a poor recurrence‐free survival in patients (Figure 7H) and *IRS1* with HR = 0.69 (0.53–0.9) and *p* = 0.0063 was related to a poor distant metastatic‐free survival (Figure 8H). Our findings concur with that mentioned in [91].

*LEP* (Leptin) is a pleiotropic molecule, which is an adipokine that enhances the survival and proliferation of breast carcinoma cells; leptin also plays a role in pathogenesis and immunological response [92, 93]. Leptin promotes metastasis and angiogenesis in breast cancer patients [94, 95]. In Figure 10I, *LEP* was discovered to have a higher promoter methylation level in the Luminal cancer subclass. The two principal mechanisms underlying the pro‐carcinogenic effect of leptin and the anti‐carcinogenic action of adiponectin are a modification of the signaling pathways that regulate the procedure of growth and development and the regulation of the apoptotic response [96]. In a study conducted, it was found that leptin promotes breast cancer bone metastasis [97]. In Figure 12I, *LEP* is positively associated with tumor purity and has a strong association with CD8+ T Cell, CD4+ T Cell, macrophage, neutrophil, and dendritic cells, thereby agreeing with the suggestion of [98]. This finding thus indicates that leptin might play a role in modulating the immune response within tumors and could thus have implications for tumor progression, immune evasion, and even therapeutic strategies.

*ADIPOQ* (adiponectin, C1Q, and collagen domain containing) is secreted by adipocytes and appears in numerous forms such as cleaved, globular isoform (gAd), and full‐length multimers (fAd) [99]. Due to *ADIPOQ* being regulated by *PPARG*, it might have a noteworthy effect on the etiology of breast cancer. It was also discovered that *ADIPOQ* increased the overall survival rate for breast cancer patients [100]; however, in this study, it was found that *ADIPOQ*, when highly expressed in breast cancer, is linked with poor recurrence‐free survival and distant metastatic‐free survival, thereby implying a greater probability of recurrence. However, the recurrence‐free survival falls in the upper quartile, with a low expression cohort of 41.39 months and a high expression cohort of 57.27 months; the distant metastatic‐free survival also falls in the upper quartile, with a low expression cohort of 236.22 months and a high expression cohort of 222.81 months; this can be due to *ADIPOQ*'s anticancer role. Prior research has indicated that *ADIPOQ* may control the processes connected to angiogenesis, tissue remodeling, and cell proliferation that are mediated by different growth factors [101].

The hub genes identified in this study were highly prevalent in 4 biological processes: glucose homeostasis, negative regulation of lipid storage, cellular response to insulin, and stimulus response to ethanol (Table 5). The genes were also predominantly enriched in 3 cellular components: extracellular space, extracellular region, and cell surface (Table 6). The top 4 molecular functions that these genes were a part of were hormone activity, cytokine activity, insulin‐like growth factor receptor binding, and insulin receptor binding (Table 7). The hub genes were predominantly enriched in the adipocytokine signaling pathway, insulin resistance, AMPK signaling pathway, and the longevity regulating pathway (Table 8).

The adipocytokine signaling pathway (Table 8) is considered a catalyst for metabolism (fatty acids) and has been shown to play a significant role in the resistance of insulin and sensitivity [102, 103]. AMPK (Table 8) is a heterotrimeric complex composed of regulatory β and γ subunits and a catalytic α subunit. Its signaling counteracts the effects of insulin and growth hormones by encouraging cells to create energy at the cost of motility and growth. However, raising AMPK activity could hinder tumor cells from metastasizing. AMPK also plays a significant part in the regulation of metabolic plasticity [104]. Moreover, aromatase is suppressed by activated AMPK, thereby reducing the production of estrogen and thus stopping the growth of breast cancer [105, 106]. A number of genes and related signaling pathways are involved in the regulation of longevity. These pathways can affect various physiological processes, including autophagy and protein synthesis [107].

In this study, 10 hub genes were identified, and 8 were found to be linked to poorer recurrence‐free survival, and 3 were identified to be linked to poor distant free‐metastatic survival. It was also found in this study that the 10 hub genes can be used as therapeutic targets and diagnostic markers for breast cancer metastasis. However, their role further needs to be explored in breast cancer brain metastasis. From this study, it can be deduced that the 10 hub genes do play a role in breast cancer brain metastasis.

Due to the complexity of the datasets that were used in this study, it was challenging to take significant characteristics like tumor staging and race into account when assessing the DEGs. In this study, the exact mechanisms of these hub genes were not identified. Therefore, further research needs to be conducted to determine the biological basis of the hub genes. This study had a small sample size, which may have biased the results. Therefore, pre‐clinical experiments and lab‐based experiments might provide additional validation of the results.

## 5 Conclusions

Using bioinformatic analysis, in this study, 4 DEGs and 10 hub genes (*IL6, INS, TNF, PPARG, PPARA, SLC2A4, PPARGC1A, IRS1, LEP*, and *ADIPOQ*) were found to be linked with the progression of breast cancer brain metastasis. The overexpression of 8 hub genes indicated a poor recurrence‐free survival, and 3 hub genes indicated a poor distant metastatic‐free survival. Further investigations and research of the identified hub genes will greatly enhance our knowledge about the progression and pathogenicity of breast cancer brain metastasis. Furthermore, the identified hub genes can serve as prospective prognostic genes and therapeutic targets for breast cancer brain metastasis. This can further be validated by pre‐clinical trials.

## Author Contributions

**Mageshree Pillay:** conceptualization (equal), data curation (equal), formal analysis (equal), investigation (equal), methodology (equal), software (equal), validation (equal), visualization (equal), writing – original draft (equal), writing – review and editing (equal). **Oliver Tendayi Zishiri:** conceptualization (equal), data curation (equal), formal analysis (equal), funding acquisition (equal), investigation (equal), methodology (equal), project administration (equal), resources (equal), software (equal), supervision (equal), validation (equal), visualization (equal), writing – original draft (equal), writing – review and editing (equal).

## Ethics Statement

The authors have nothing to report.

## Conflicts of Interest

The authors declare no conflicts of interest.

## Data Availability

The datasets used in this study are available from the GEO database.
